# Trends in *Clostridioides difficile* prevalence, mortality, severity, and age composition during 2003–2014, the national inpatient sample database in the US

**DOI:** 10.1080/07853890.2022.2092893

**Published:** 2022-07-04

**Authors:** Sun O. Park, Ilhwan Yeo

**Affiliations:** a Division of Infectious Diseases, Department of Medicine, Albert Einstein College of Medicine and Montefiore Medical Center, Bronx, NY, USA; b Department of Medicine, New York Presbyterian, Queens, NY, USA

**Keywords:** *Clostridium difficile*, *Clostridioides difficile*, trends, mortality, severity, age composition

## Abstract

**Background:**

*Clostridioides difficile* (formerly known as *Clostridium difficile*) infection (CDI) is one of the most prevalent healthcare-associated infections in the United States (US). In the early 2000s, CDI emerged as a great threat with increasing prevalence, mortality, and severity, especially in advanced age. We investigated the US national trends in in-hospital CDI prevalence, mortality, severity, and age composition from 2003 to 2014.

**Methods:**

We identified the patients with CDI using the national inpatient sample data from 2003 to 2014. We performed Poisson regression model and Kendall’s tau-b correlation test for our analyses.

**Results:**

Adjusted overall CDI prevalence did not significantly change during 2003–2014. In-hospital mortality of overall CDI did not significantly change during 2003–2008, then significantly decreased during 2008–2014. Severity of overall CDI significantly increased during 2003–2008, then decreased during 2008–2014. The proportions of patients with age ≥ 65 years decreased in CDI prevalence, mortality, and severity during 2003–2014.

**Conclusions:**

Compared to the earlier years 2003–2008, overall CDI outcome improved in the later years 2008–2014. Younger patients increasingly contributed to CDI prevalence, mortality, and severity during 2003–2014. More studies to understand underlying driving forces of changes in CDI trends are warranted to mitigate CDI.

## Introduction

*Clostridium difficile* established itself as a pathogen of hospital-acquired infection by the 1990s [1]. Before 2000, CDI was considered manageable other than occasional outbreaks and an issue of recurrence of disease. In the early 2000s, CDI was significantly increased in the prevalence, mortality, and severity in North America and Europe, associated with emergence of the fluoroquinolone-resistant, hypervirulent strain, NAP1/BI/027 [2–4]. Since then, CDI has become one of the most important hospital-acquired pathogens.

Studies using national (year 2004–2014) or Veterans health database (year 2006–2016) showed increasing trends in CDI cases while decreasing trends in CDI associated mortality [5–7]. Previous studies using the National Inpatient Sample (NIS) database limited the analyses to the primary diagnoses of CDI or the patients in certain age range or did not adjust the rates for confounding factors [5,6].

To estimate national burden of in-hospital CDI in the US since the early 2000s, we investigated 12-year trends in CDI prevalence, mortality, and severity from 2003 to 2014 using the National Inpatient Sample (NIS) database. It is known that CDI disproportionately attack persons with advanced age [2,8]. We investigated changes in age composition of CDI prevalence, CDI-associated mortality, and severe CDI.

## Materials and methods

### Data source

We used the NIS database, developed as part of Healthcare Cost and Utilization Project (HCUP) sponsored by the Agency for Healthcare Research and Quality (AHRQ). The NIS, a large publicly available all-payer inpatient heath care database, was sampled from the State In patient Database by systemic sampling design, approximating a 20% stratified sample of discharges from the US community hospitals (https://www.hcup-us.ahrq.gov/nisoverview.jsp). Each observation in NIS, a unique discharge record, includes each discharged person’s primary and secondary diagnoses and procedures performed during the index hospitalization, indicated by International Classification of Diseases-Ninth Revision-Clinical Modification (ICD-9-CM) codes. Each record also includes demographics, comorbidities, All Patient Refined Diagnosis-Related Groups (APR-DRG) severity of illness, APR-DRG risk of mortality, length of hospital stay (LOS), hospital location/teaching status, hospital region, estimated median household income quartile based on the patient’s zip code, total hospital charge, primary payer, discharge disposition, and in-hospital mortality. We received an exemption from the Albert Einstein College of Medicine Institutional Review Board because the NIS data were deidentified.

### Study design and definitions

We performed a serial retrospective cross-sectional study of the patients discharged from the US hospitals using the NIS database from 2003 to 2014.

Primary diagnosis is defined as the condition chiefly responsible for the patient’s hospitalization. Secondary diagnoses (up to 30 during the study period) are additional conditions that contributed to the patient’s hospitalization. The patients with primary or secondary diagnosis of CDI were identified with ICD-9-CM code 008.45. If the patients had both primary and secondary CDI for the same discharge record, only primary CDI was counted. We defined overall CDI as primary and secondary CDI combined. There is no consensus to define severe CDI. CDI with toxic megacolon, perforated colon, total abdominal colectomy (TAC), shock requiring vasopressor therapy, or death was previously described as complicated or severe CDI [9,10]. Information about vasopressors usage or stays in intensive care units was not available in the NIS database. We defined severe CDI as CDI with toxic megacolon (ICD-9-CM code 558.2), total abdominal colectomy (45.8, 45.81, 45.82, 45.83), or perforated colon (569.83). Each record has 29 binary comorbidity variables, which were developed by Elixhauser et al. [11]. We defined Elixhauser comorbidity index as the sum of each comorbidity present. APR-DRG (3M Health Information Systems, Wallingford, CT) is an expansion of the basic DRG, where each APR-DRG has four severity of illness subclasses and four risk of mortality subclasses. Subclasses are numbered from 1 to 4 indicating respectively minor, moderate, major, and extreme severity of illness or risk of mortality.

### Statistical analysis

To compare the entire NIS population with overall CDI, and primary with secondary CDI in various clinical characteristics, we performed bivariate analyses using Chi-square test for categorical variables and Student *t*-test or Mann–Whitney–Wilcoxon nonparametric test for continuous variables, dependent on the validity of normality assumption.

We determined CDI prevalence as counts of CDI diagnoses divided by counts of total discharges. We determined CDI-associated in-hospital mortality as counts of expired patients divided by counts of CDI diagnoses. We determined severe CDI rates as counts of severe CDI divided by counts of CDI diagnoses. All the rates were then calculated per 1000 discharges. Age-stratified CDI prevalence, mortality, and severity were also calculated.

Prevalence ratios (PRs), ratios of the prevalence, determined the changes in prevalence in reference to year 2003.

Prior to year 2012, the NIS database was constructed from a sample of hospitals from which all discharges were retained. Beginning of year 2012, the NIS database was constructed from a systemic sample of discharge records from all HCUP-participating hospitals rather than a sample of hospitals to improve national estimates. As instructed by AHRQ (https://www.hcup-us.ahrq.gov/db/nation/nis/trendwghts.jsp), we used the new discharge trend weight from 2003 to 2011 that were developed by AHRQ to reflect above sampling method change, and the regular discharge weight from 2012 to 2014.

We performed Poisson regression analyses to determine annual CDI prevalence, mortality, and severity. We adjusted for age, gender, race, and comorbidities using multiple Poisson regression model. We used Kendall’s tau-b (*τ*_b_) to determine strength and direction of trends in annual CDI prevalence, mortality, and severity [12].

We accounted for complex survey design of the NIS for the analyses. We performed all statistical analyses using SAS software, version 9.4 (SAS Institute, Inc., Cary, NC). A two-sided *p*-value of < .05 was considered statistically significant.

## Results

### Clinical characteristics of CDI patients

We identified a total of 3,739,014 *C. difficile* infections with ICD-9-CM code from 2003 to 2014 (Figure S1). Table 1 shows clinical characteristics of the entire NIS patients and CDI patients. *P*-values for comparisons of the entire NIS patients with overall CDI, and primary CDI with secondary CDI patients were all significant. CDI patients were older than the entire NIS patients (mean age 67 versus 48). Sixty four percent of CDI patients and 34.4% of the entire NIS patients were age 65 years or older. CDI significantly affected women (58.2%), whites (64.46%), and medicare patients (67.7%). Compared to the entire NIS patients, CDI patients had higher Elixhauser comorbidity index, APR-DRG severity of illness, APR-DRG risk of mortality, LOS, hospitalization charges, discharge to long-term care facilities, and in-hospital death. Comparing primary with secondary CDI, the patients with secondary CDI had higher Elixhauser comorbidity index, APR-DRG severity of illness, APR-DRG risk of mortality, LOS, hospitalization charges, discharge to long-term care facilities, and in-hospital death.

**Table 1.** Clinical characteristics of NIS versus overall CDI and primary versus secondary CDI.

Characteristics *n*: weighted number	NIS	Overall CDI	Primary CDI	Secondary CDI	*p*-value
	(*n* = 446,348,443)	(*n* = 3,739,014)	(*n* = 1,144,341)	(*n* = 2,594,673)	
Age, year					
Mean ± SE (median)	48.1 ± 0.2	67.3 ± 0.2	67.3 ± 0.1	67.3 ± 0.2	<.001
	(51.6)	(71.4)	(71.9)	(71.2)	
Age group, %					<.001
≤ 18	17.1	2.8	3.0	2.8	
19–44	24.8	9.3	10.2	8.9	
45–64	23.7	23.7	22.5	24.3	
65–79	20.2	32.7	31.2	33.3	
≥ 80	14.2	31.5	33.1	30.7	
Sex, %					<.001
Female	41.7	58.2	64.7	55.3	
Male	58.3	41.8	35.3	44.7	
Race, %					
White	54.5	64.6	68.0	63.1	<.001
Black	11.8	10.3	8.0	11.2	
Hispanic	10.5	6.7	6.4	6.8	
Asian	2.2	1.6	1.1	1.8	
Native American	0.6	0.5	0.4	0.5	
Other	2.8	2.0	1.8	2.2	
Missing	17.7	14.4	14.3	14.4	
Elixhauser comorbidity index					
Mean ± SE (median)	1.8 ± 0.0 (0.9)	3.5 ± 0.0 (2.9)	3.4 ± 0.0 (2.8)	3.6 ± 0.0 (2.9)	<.001
APR-DRG severity of illness					
Mean ± SE (median)	1.9 ± 0.0 (1.3)	3.1 ± 0.0 (2.7)	2.6 ± 0.0 (2.1)	3.4 ± 0.0 (2.9)	<.001
APR-DRG risk of mortality					
Mean ± SE (median)	1.6 ± 0.0 (0.8)	2.6 ± 0.0 (2.1)	2.1 ± 0.0 (1.6)	2.8 ± 0.0 (2.4)	<.001
Length of hospital stay					
Mean ± SE (median)	4.6 ± 0.0 (2.4)	11.8 ± 0.1 (7.0)	6.6 ± 0.0 (4.4)	14.1 ± 0.1 (8.9)	<.001
Mean ± SE (median)^a^	4.5 ± 0.0 (2.4)	11.4 ± 0.1 (6.9)	6.5 ± 0.0 (4.4)	13.8 ± 0.1 (8.9)	
Hospital location/teaching status, %					<.001
Rural	12.3	9.7	12.7	8.4	
Urban non-teaching	40.0	39.8	43.5	38.2	
Urban teaching	47.7	50.5	43.8	53.4	
Hospital region, %					
Northeast	19.3	23.3	22.1	23.8	<.001
Midwest	22.8	24.8	25.0	24.7	
South	38.4	34.2	36.7	33.2	
West	19.5	17.7	16.2	18.3	
Median household income, %					
1st quartile	29.0	25.8	25.3	26.1	<.001
2nd quartile	26.0	25.2	25.8	24.7	
3rd quartile	23.7	24.9	25.1	24.9	
4th quartile	21.3	24.1	23.8	24.3	
Hospitalization charges, $					
Mean ± SE	32,900 ± 5,729	82,773 ± 9,523	35,131 ± 2,486	103,991 ± 15,327	<.001
(median)	(17,466)	(38,104)	(21,480)	(51,709)	
Mean ± SE^a^	31,872 ± 5,661	76,950 ± 8,913	33,799 ± 2,728	97,772 ± 14,520	
(median)	(17,273)	(36,514)	(19,310)	(49,850)	
Primary payer, %					
Medicare	37.7	67.7	67.3	67.9	<.001
Medicaid	20.0	9.3	7.9	10.0	
Private	33.4	18.6	20.4	17.8	
Other	8.9	4.4	4.4	4.3	
Discharge disposition %					<.001
Home	82.2	50.7	66.1	43.9	
Transfer to acute care	2.1	2.6	1.3	3.1	
Long-term care	12.7	37.9	29.1	41.8	
AMA or unknown	0.9	0.4	0.4	0.5	
Died	2.0	8.3	3.0	10.6	

NIS: national inpatient sample; CDI: *Clostridioides difficile*; AMA: against medical advice; CDI: *Clostridioides difficile* infection; PR: prevalence ratio.

Elixhauser comorbidity index is the sum of 29 binary comorbidity variables present. APR-DRG has four subclasses (1 – minor, 2 – moderate, 3 – major, 4 – extreme). Hospitalization charges (the dollar amount that a hospital sets for services before negotiating any discounts) were adjusted for inflation with 2014 as standard year. 95% confidence intervals are in parenthesis.

^a^Only survivors were included. *p*-values represent comparisons between NIS and overall CDI or between primary CDI and secondary CDI patients.

### Trends in annual CDI prevalence

The crude prevalence of primary CDI significantly increased from 1.3 to 3.0 per 1000 discharges from 2003 to 2014 (*τ_b_*=0.84, *p* < .001). The crude prevalence of secondary CDI significantly increased from 4.1 to 7.2 per 1000 discharges from 2003 to 2014 (*τ_b_*=0.83, *p* < .001). Consequently, the crude prevalence of overall CDI significantly increased from 2003 to 2014 (*τ_b_*=0.97, *p* < .001). There were no significant changes in adjusted CDI prevalence (primary, secondary, and overall) from 2003 to 2014 (*p* > .05 for trends in adjusted PRs), other than significant upward trends in primary CDI prevalence from 2003 to 2008 (*τ_b_*≈1, *p* = .0048) (Table 2).

**Table 2.** Annual trends in unadjusted and adjusted prevalence of CDI.

	Overall CDI		Primary CDI		Secondary CDI	
Year	Unadjusted PR	Adjusted PR	Unadjusted PR	Adjusted PR	Unadjusted PR	Adjusted PR
2003	1	1	1	1	1	1
2004	1.16	1.16	1.22	1.21	1.15	1.15
	(1.16–1.17)	(1.16–1.17)	(1.20–1.23)	(1.20–1.23)	(1.14–1.15)	(1.14–1.16)
2005	1.39	1.33	1.52	1.43	1.36	1.30
	(1.39–1.40)	(1.33–1.34)	(1.50–1.54)	(1.42–1.45)	(1.35–1.36)	(1.29–1.31)
2006	1.45	1.32	1.78	1.61	1.35	1.23
	(1.44–1.46)	(1.31–1.32)	(1.76–1.80)	(1.59–1.63)	(1.34–1.36)	(1.22–1.24)
2007	1.49	1.32	2.05	1.81	1.32	1.16
	(1.49–1.50)	(1.31–1.32)	(2.03–2.08)	(1.79–1.84)	(1.31–1.33)	(1.15–1.17)
2008	1.57	1.28	2.22	1.82	1.37	1.11
	(1.56–1.58)	(1.27–1.29)	(2.20–2.25)	(1.80–1.84)	(1.36–1.38)	(1.10–1.12)
2009	1.53	1.21	2.19	1.74	1.33	1.04
	(1.52–1.54)	(1.20–1.21)	(2.16–2.21)	(1.72–1.76)	(1.32–1.34)	(1.03–1.05)
2010	1.60	1.21	2.24	1.72	1.40	1.05
	(1.59–1.61)	(1.20–1.22)	(2.22–2.27)	(1.71–1.74)	(1.39–1.41)	(1.04–1.05)
2011	1.80	1.23	2.51	1.77	1.58	1.06
	(1.79–1.81)	(1.23–1.24)	(2.49–2.54)	(1.75–1.79)	(1.57–1.59)	(1.05–1.07)
2012	1.83	1.27	2.56	1.81	1.61	1.09
	(1.82–1.84)	(1.26–1.27)	(2.53–2.59)	(1.79–1.83)	(1.60–1.62)	(1.09–1.10)
2013	1.85	1.24	2.45	1.68	1.67	1.10
	(1.84–1.86)	(1.24–1.25)	(2.42–2.47)	(1.66–1.70)	(1.66–1.68)	(1.09–1.11)
2014	1.89	1.24	2.38	1.61	1.73	1.11
	(1.88–1.90)	(1.23–1.24)	(2.36–2.41)	(1.59–1.63)	(1.72–1.74)	(1.10–1.12)

CDI prevalence of overall CDI age group G0 (age 0–18), G1 (age 19–44), G2 (age 45–64), G3 (age 65–79), and G4 (age ≥ 80 years) were 0.14%, 0.31%, 0.84%, 1.35%, and 1.85%, respectively. There were downward trends in proportions of age ≥ 65 years among CDI patients during 2003–2014 (*τ*_b_= −0.81, *p* = .0003); 67.3% and 58.3% of overall CDI were age ≥ 65 years in 2003 and 2014, respectively. The greatest increase in the age composition of overall CDI was the age group G2 (6.9% increase) while the greatest decrease was the age group G4 (6% decrease) during 2003–2014 (Figure S2a and S2b).

### Trends in annual in-hospital mortality

In-hospital crude mortality rate (CMR) of primary CDI significantly decreased from 38.2 to 16.7 per 1000 primary CDIs from 2003 to 2014 (*τ*_b_= −0.70, *p* = .002). In-hospital CMR of secondary CDI significantly increased from 108.8 to 122.7 per 1000 secondary CDIs from 2003 to 2008 (*τ_b_*=0.87, *p* = .01), then significantly decreased from 122.7 to 88.1 from 2008 to 2014 (*τ*_b_≈−1.0, *p* = .0016). While there were no significant changes from 2003 to 2008, in-hospital CMR of overall CDI significantly decreased from 94.8 to 66.9 per 1000 overall CDIs from 2008 to 2014 (*τ*_b_≈−1.0, *p* = .0016). Adjusted in-hospital mortality of primary CDI significantly decreased by 55% from 2003 to 2014 (*τ*_b_=−0.81, *p* < .001). Adjusted in-hospital mortality of secondary and overall CDI significantly decreased by 30% and 28%, respectively, from 2008 to 2014 (*τ*_b_≈−1.0, *p* = .001 for both) while there were no significant changes from 2003 to 2008 (Table 3).

**Table 3.** Annual trends in unadjusted and adjusted mortality rates of CDI.

	Overall CDI		Primary CDI		Secondary CDI	
Year	Unadjusted IRR	Adjusted IRR	Unadjusted IRR	Adjusted IRR	Unadjusted IRR	Adjusted IRR
2003	1	1	1	1	1	1
2004	1.00	0.97	0.94	0.90	1.02	0.98
	(0.98–1.02)	(0.95–0.99)	(0.88–1.00)	(0.84–0.96)	(1.00–1.04)	(0.96–1.00)
2005	1.01	0.96	1.05	0.99	1.02	0.96
	(0.99–1.02)	(0.94–0.97)	(0.99–1.12)	(0.93–1.05)	(1.00–1.04)	(0.94–0.98)
2006	0.97	0.90	0.90	0.82	1.03	0.95
	(0.95–0.99)	(0.88–0.92)	(0.85–0.96)	(0.77–0.87)	(1.01–1.05)	(0.93–0.97)
2007	0.99	0.90	0.99	0.86	1.07	0.98
	(0.97–1.01)	(0.88–0.92)	(0.94–1.05)	(0.81–0.91)	(1.05–1.09)	(0.96–1.00)
2008	1.03	0.97	1.02	0.93	1.13	1.06
	(1.01–1.05)	(0.95–0.99)	(0.97–1.08)	(0.88–0.99)	(1.11–1.15)	(1.04–1.08)
2009	0.97	0.90	0.95	0.86	1.07	0.99
	(0.95–0.99)	(0.89–0.92)	(0.90–1.00)	(0.81–0.91)	(1.05–1.09)	(0.97–1.01)
2010	0.89	0.83	0.81	0.75	0.98	0.91
	(0.87–0.90)	(0.81–0.85)	(0.76–0.86)	(0.71–0.80)	(0.96–1.00)	(0.89–0.93)
2011	0.83	0.76	0.68	0.63	0.93	0.84
	(0.81–0.84)	(0.75–0.78)	(0.64–0.72)	(0.59–0.67)	(0.91–0.95)	(0.83–0.86)
2012	0.78	0.74	0.58	0.56	0.88	0.82
	(0.76–0.79)	(0.72–0.75)	(0.54–0.61)	(0.52–0.59)	(0.86–0.90)	(0.80–0.84)
2013	0.77	0.73	0.53	0.52	0.86	0.80
	(0.76–0.78)	(0.71–0.74)	(0.50–0.57)	(0.49–0.56)	(0.85–0.88)	(0.79–0.82)
2014	0.73	0.69	0.44	0.45	0.81	0.76
	(0.71–0.74)	(0.68–0.71)	(0.41–0.47)	(0.42–0.48)	(0.79–0.83)	(0.74–0.77)

*Note*: 95% confidence intervals are in parenthesis. CDI: *Clostridioides difficile* infection; IRR: incidence rate ratio.

In-hospital mortality of overall CDI age group G0, G1, G2, G3, and G4 were 1.5%, 2.9%, 6.0%, 8.9%, and 11.6%, respectively. There were downward trends in proportions of age ≥ 65 years among overall CDI-associated in-hospital deaths during 2003–2014 (*τ*_b_= −0.72, *p* = .0012); 79.8% and 73.3% of overall CDI-associated in-hospital deaths were age ≥ 65 years in 2003 and 2014, respectively. The greatest increase in the age composition of the dead among overall CDIs occurred in the age group G2 (6.1% increase) while the greatest decrease occurred in the age group G4 (5.5% decrease) during 2003–2014 (Figure S2c and S2d).

### Trends in annual severe CDI

Severe overall CDI consisted of TAC (50.1%), perforated colon (39.1%), toxic megacolon (5.1%), and more than one of these conditions (5.7%). The crude prevalence of severe primary CDI significantly decreased from 7.7 to 3.0 per 1000 primary CDIs from 2003 to 2014 (*τ*_b_=−0.85, *p* = .001). The crude prevalence of severe secondary CDI significantly increased from 7.4 to 11.7 per 1000 secondary CDIs from 2003 to 2008 (*τ_b_* = 0.83, *p* = .02), then decreased from 11.7 to 10.4 from 2008 to 2014 (*τ*_b_ = −0.49, *p* = .13). The crude prevalence of severe overall CDI significantly increased from 7.5 to 9.9 per 1000 overall CDIs from 2003 to 2008 (*τ_b_* = 0.73, *p* = .04), then decreased from 9.9 to 8.2 from 2008 to 2014 (*τ*_b_=−0.71, *p* = .02).

Downward trends in adjusted rates of severe primary CDI during 2003–2014 remained significant with rate reduction of 58% (*τ*_b_=−0.85, *p* = .0001). Trends in adjusted rates of severe secondary CDI increased by 47% from 2003 to 2008 (*τ_b_* = 0.73, *p* = .04), then decreased by 21% from 2008 to 2014 (*τ*_b_ = −0.65, *p* = .046). Adjusted rates of severe overall CDI increased by 26% from 2003 to 2008 (*τ_b_* = 0.73, *p* = 0.04), then decreased by 24% from 2008 to 2014 (*τ*_b_ = −0.81, *p* = .01) (Table 4).

**Table 4.** Annual trends in unadjusted and adjusted rates of severe CDI.

	Overall CDI		Primary CDI		Secondary CDI	
Year	Unadjusted PR	Adjusted PR	Unadjusted PR	Adjusted PR	Unadjusted PR	Adjusted PR
2003	1	1	1	1	1	1
2004	0.89	0.87	0.89	0.90	0.89	0.86
	(0.83–0.95)	(0.81–0.94)	(0.77–1.02)	(0.78–1.04)	(0.82–0.96)	(0.79–0.94)
2005	0.96	0.94	0.86	0.87	1.00	0.96
	(0.90–1.03)	(0.88–1.00)	(0.75–0.99)	(0.76–1.01)	(0.92–1.07)	(0.89–1.04)
2006	1.02	1.01	0.91	0.95	1.07	1.05
	(0.96–1.09)	(0.95–1.08)	(0.79–1.03)	(0.83–1.08)	(1.00–1.16)	(0.97–1.13)
2007	1.04	1.02	0.71	0.72	1.20	1.16
	(0.98–1.11)	(0.95–1.09)	(0.62–0.81)	(0.63–0.83)	(1.12–1.30)	(1.08–1.25)
2008	1.32	1.26	0.83	0.86	1.57	1.47
	(1.25–1.41)	(1.19–1.34)	(0.73–0.94)	(0.75–0.98)	(1.47–1.68)	(1.37–1.58)
2009	1.30	1.21	0.79	0.82	1.56	1.42
	(1.22–1.38)	(1.14–1.29)	(0.69–0.90)	(0.72–0.94)	(1.45–1.67)	(1.32–1.53)
2010	1.34	1.25	0.76	0.79	1.63	1.48
	(1.26–1.43)	(1.17–1.33)	(0.67–0.87)	(0.69–0.90)	(1.52–1.75)	(1.38–1.59)
2011	1.25	1.16	0.61	0.63	1.57	1.42
	(1.18–1.33)	(1.09–1.23)	(0.53–0.69)	(0.55–0.72)	(1.47–1.69)	(1.33–1.53)
2012	1.11	1.04	0.55(0.48–0.6	0.58	1.39	1.26
	(1.04–1.18)	(0.97–1.11)	3)	(0.51–0.67)	(1.29–1.49)	(1.17–1.36)
2013	1.06	0.98	0.49	0.52	1.32	1.20
	(1.00–1.13)	(0.92–1.05)	(0.42–0.56)	(0.45–0.60)	(1.23–1.42)	(1.11–1.29)
2014	1.09	1.02	0.39	0.42	1.40	1.26
	(1.03–1.16)	(0.95–1.08)	(0.33–0.45)	(0.36–0.49)	(1.31–1.50)	(1.18–1.36)

95% confidence intervals are in parenthesis. CDI: *Clostridioides difficile* infection; PR: prevalence ratio.

Severe CDIs among overall CDI age group G0, G1, G2, G3, and G4 were 0.49%, 0.77%, 0.96%, 1.03%, and 0.62%, respectively. There were downward trends in proportions of age ≥ 65 years among severe overall CDIs during 2003–2014 while not statistically significant (*τ*_b_= −0.39, *p* = .07); 64.3% and 54.5% of overall CDIs were age ≥ 65 years in 2003 and 2014, respectively. The greatest increase in the age composition of severe overall CDIs occurred in the age group G2 (6.7% increase) while the greatest decrease in the age composition occurred in the age group G4 (5% decrease) during 2003–2014 (Figure S2e and S2f).

## Discussion

Epidemiology of CDI is continually evolving. CDI prevalence, severity, and mortality increased during 1991–2003 [9, 10]. A study using NIS database showed a 109% increase in CDI prevalence, an 18% increase in mortality, and a 183% increase in colectomy among CDI patients from 1993 to 2003 [10]. A retrospective study at a medical centre in Quebec demonstrated a 4.4-fold increase in CDI incidence (an 8.5-fold among age ≥ 65 years), a 2.6-fold increase in complicated CDI, and a 3-fold increase in CDI-associated mortality from 1991 to 2003 [9].

Using a nationally representative database of the US hospitals, we demonstrated that primary CDI trended upwards in prevalence and trended downwards in mortality and severity while secondary CDIs trended upwards in severity without significant changes in prevalence and mortality from 2003 to 2008. Compared to the patients with secondary CDIs, the patients with primary CDIs were less sick, illustrated by lower comorbidity index and APR-DRG scores, and more likely community-acquired infections [5,13]. Possibly there was a significant rise in community-acquired CDIs that might have led to increase in primary CDI prevalence but decrease in primary CDI mortality and severity during 2003–2008 by capturing relatively healthy patients who were less likely to become critically ill or die. Despite no significant changes in secondary CDI-associated mortality from 2003 to 2008, secondary CDI severity increased. Overall improvement of mortality in hospitalized patients might have offset possible upward trends in secondary CDI-associated mortality from 2003 to 2008. Our study demonstrated improved outcomes of CDIs with decreased mortality and severity from 2008 to 2014, similar to the published studies [5–7]. A population-based surveillance program from the Centres for Disease Control and Prevention (CDC) showed that the adjusted estimate of total national burden of CDI decreased annually by 4% while no significant changes in in-hospital deaths during 2011–2017 [14]. However, this study likely underestimated CDI cases by fixing NAAT use at the 2011 rate, which could have biased in-hospital death rates as well.

Significant improvement of CDI outcomes during 2008–2014 might be attributable to the changes in CDI strains. Significant rise in CDI cases, transmission, and severity, and CDI-associated mortality in North America and Europe in the early 2000s were attributable to the emergence of the hypervirulent BI/NAP1/027 [2–4,15,16]. The NAP1/BI/027 strain accounted for 36% of *C. difficile* isolates from the patients in North America enrolled in clinical trials during 2005–2007 [17]. In more recent years, there were downward trends in the prevalence of the NAP1/BI/027 strain. The prevalence of the NAP1/BI/027 strain during 2012–2016 was 22% across the US Veterans Health Administration with significant downward trends (16.9% in 2016) [18]. A US national surveillance program from 6 geographically distinct medical centres showed that ribotype 027 markedly declined from 35.3% in 2011 to 13.1% in 2016 and ribotype 106 became the most common type (15.0%), followed by ribotypes 027 and 014/020 in 2016 [19]. A study involved in 26 laboratories in the US during 2011–2017 showed similar results [20]. Likewise, the prevalence of the NAP1/BI/027 strain has markedly decreased since 2007 with increasing diversity of strains in the UK [3].

Over-testing for *C. difficile* due to heightened public awareness and use of sensitive molecular test can spuriously increase CDI prevalence but decrease CDI mortality and severity. On the other hand, under-testing can potentially decrease CDI cases but increase CDI mortality and severity by selecting sicker patients. Nucleic acid amplification tests (NAATs) are rapid and highly sensitive and specific for detecting the presence of toxin-producing genes. However, NAATs can’t differentiate *C. difficile* colonization from active disease [3,16,21]. Since NAATs became available in 2009 in the US, many laboratories switched toxin tests to NAATs to increase sensitivity, which could have led to overdiagnoses of CDIs by detecting asymptomatic carriage [21]. Some centres reported increased CDI rates by 50–100% after switching to NAATs [3]. An analysis of population-based surveillance data from 3 US states during 2009–2011 demonstrated that switching from toxin enzyme immunoassay tests to NAATs increased CDI cases by 43–67% and test positivity rate from 10.4% to 19.4% [22]. A study from the Duke Infection Control Outreach Network hospitals demonstrated that the average increase in rate after switching to molecular testing was 56% during July 2009 − December 2011 [23]. Despite availability of NAATs in 2009, no significant upward trends in CDI prevalence after 2009 in our study indicate that other factors such as changes in CDI strains might have negated changes from NAATs use. Additionally, adjustment of potential confounding factors such as comorbidities might have corrected inflated CDI rates from NAATs use.

Advanced age is known to be an important risk factor for CDI and severe CDI [2]. Increased trends in CDI cases were prominent among age ≥ 65 years in early to mid-2000s [24]. Our study illustrated that there were downward trends in proportions of age ≥ 65 years in CDI prevalence, mortality, and severity during 2003–2014. Consequently, the proportions of younger patients increased in CDI prevalence, mortality, and severity during 2003–2014 with the greatest increase in age 19–44 years.

## Limitations

Our study has limitations inherent to the retrospective study of the administrative database. (1) We could not differentiate between *C. difficile* colonization and clinical infection. We determined CDI diagnoses based on ICD-9-CM codes without verifying with laboratory test results as laboratory data are not available in the NIS database. However, previous studies showed good correlation between CDI diagnoses and ICD-9-CM coding [5,25]. (2) Each record in the NIS is for a single hospitalization, not a person. Therefore, one person can be counted multiple times depending on the number of admissions during the study period. (3) Whether death, perforation, or colectomy was caused by CDI could not be determined using our data. (5) Information on *C. difficile* strains and types of laboratory methods used for diagnoses of CDIs was not available. (6) It is possible that we did not account for important confounding factors. (7) While TAC has been considered the surgical standard of care for complicated CDI, loop ileostomy with colonic lavage has been increasingly performed as an alternative since publication by Neal et al. in 2011 [26]. Omission of LI with colonic lavage in this study might have underestimated severe CDI after 2011, however, inclusion of less invasive and organ preserving nature of this new approach could have selected non-severe CDI patients. (8) The patients with poor surgical candidacy might have been excluded from severe CDIs.

## Conclusions

In summary, epidemiology of CDI is dynamic with highly adaptive nature of *C. difficile*. From 2003 to 2008, overall CDI prevalence and mortality didn’t show significant changes while severity increased. From 2008 to 2014, overall CDI mortality and severity significantly decreased without significant changes in prevalence. To our knowledge, our study is the first to show that the proportions of age ≥ 65 years decreased in CDI prevalence, mortality, and severity from 2003 to 2014 using the US national data. Transformation of CDI epidemiology must be attributable to multiple factors, including changes in *C. difficile* strains, testing strategy, treatment method, antibiotic use pattern, and changes in infection control strategies. Surveillance of *C. difficile* strains and understanding of CDI epidemiology are essential to guide us to prevent serious *C. difficile* outbreaks or surge in the future.

## Supplementary Material

Supplemental Material

## Data Availability

National Inpatient Sample (NIS) data acquired from Healthcare Cost and Utilization Project (HCUP), Agency for Healthcare Research and Quality (AHRQ), Rockville, MD (https://www.hcup-us.ahrq.gov/nisoverview.jsp) are available to the researchers following a standard application process and signing of a data use agreement.
