# Supplementary material for: Trends in Clostridioides difficile prevalence, mortality, severity, and age composition during 2003–2014, the national inpatient sample database in the US
Source: Ann Med. 2022 Jul 4;54(1):1851–8. doi: 10.1080/07853890.2022.2092893 (PMC9258430; doi:10.1080/07853890.2022.2092893)
Supplement: Supplemental Material [file IANN_A_2092893_SM2498.zip › Supplemental files/AnnOfMedSupplementary Figure legend_3_17_22.docx]

**Supplementary Figures legend**

**Figure S1. Population derivation chart.** Abbreviations: NIS, national inpatient sample; N, number; ICD-9-CM, International Classification of Diseases-Ninth Revision-Clinical Modification (ICD-9-CM).

**Figure S2. Trends in *Clostridioides difficile* Prevalence, Mortality, Severity by age group.** (a) Overall CDI cases per 1000 discharges, (c) Deaths per 1000 overall CDI cases, (e) Severe CDI cases per 1000 overall CDI cases are presented annually from 2003 to 2014 by age group. (b) Age compositions of overall CDI cases (d) Age compositions of dead among overall CDI cases (f) Age compositions of severe overall CDI cases are presented annually from 2003 to 2014. Abbreviations: CDI, *C. difficile* infection; G0, age 0−18 years; G1, age 19−44 years; G2, age 45−64 years; G3, age 65−79 years; G4, age $\geq$ 80 years.
