# Supplementary figures and images for: Trends in Clostridioides difficile prevalence, mortality, severity, and age composition during 2003–2014, the national inpatient sample database in the US
Source: Ann Med. 2022 Jul 4;54(1):1851–8. doi: 10.1080/07853890.2022.2092893 (PMC9258430; doi:10.1080/07853890.2022.2092893)

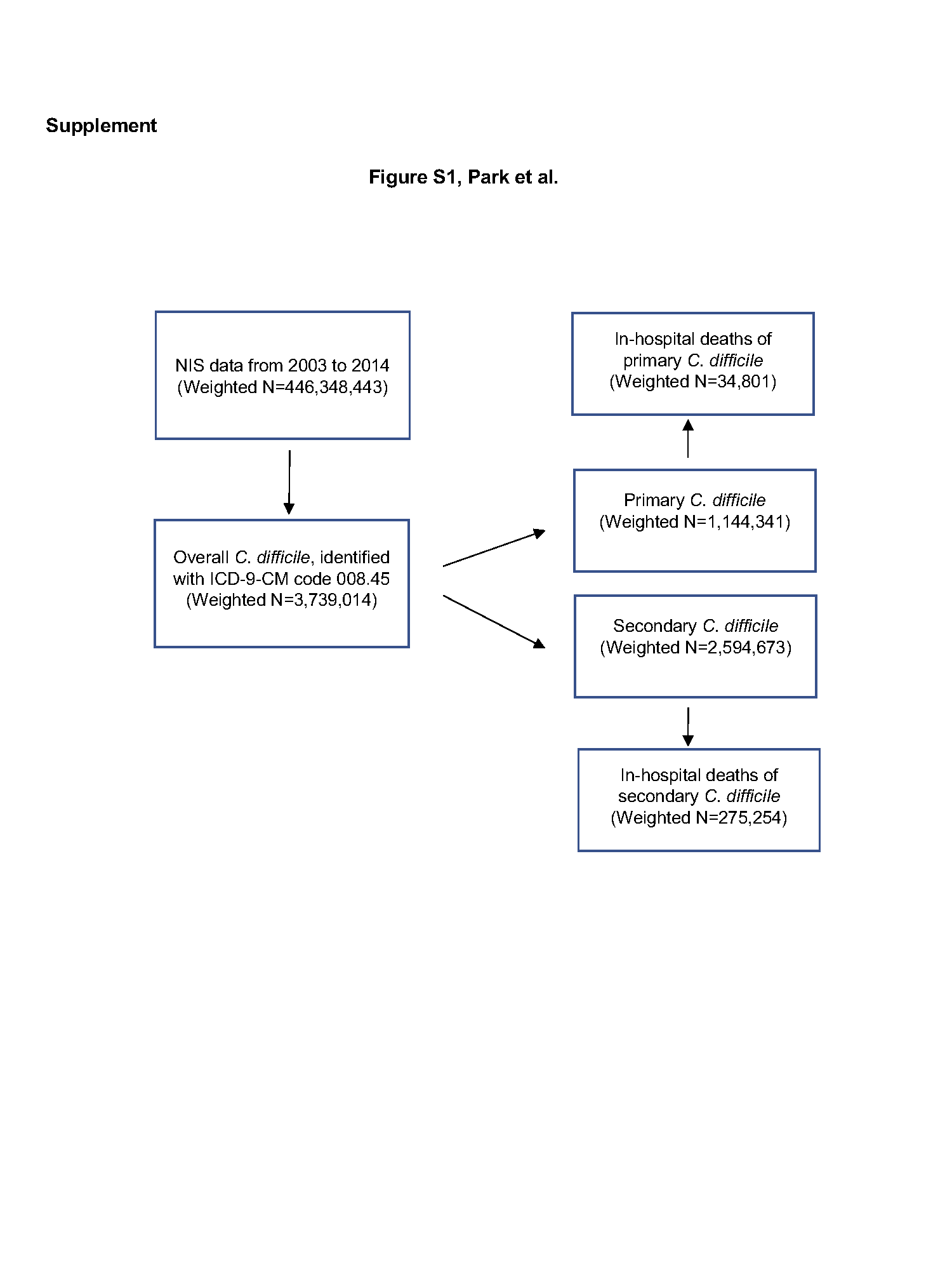

Supplement: Supplemental Material [file IANN_A_2092893_SM2498.zip › Supplemental files/AnnOfMedSupplement Supp_Figure 1_3_17_22_Page_1.tiff]

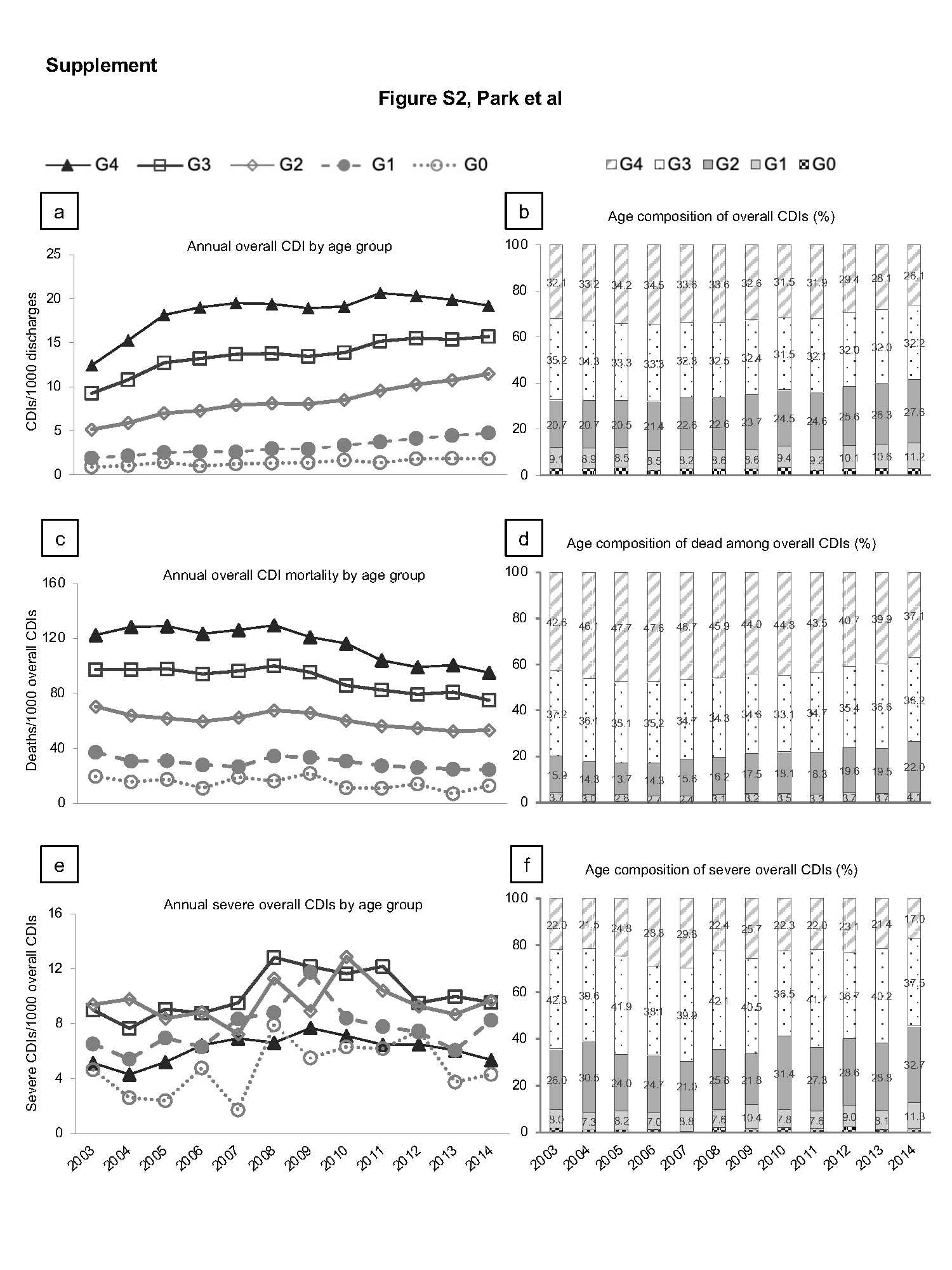

Supplement: Supplemental Material [file IANN_A_2092893_SM2498.zip › Supplemental files/AnnOfMedSupplement Supp_Figure 2_3_17_22_Page_2.tiff]
